# Comparative transcriptomic analysis and genome-wide identification provide insights into the potential role of fungal-responsive MAPK cascade genes in tanshinone accumulation in *Salvia miltiorrohiza*

**DOI:** 10.3389/fpls.2025.1583953

**Published:** 2025-05-16

**Authors:** Ann Abozeid, Xinru Du, Lan Zhang, Furui Yang, Jianxiong Wu, Lin Zhang, Qi Cui, Zongqi Yang, Dongfeng Yang

**Affiliations:** ^1^ College of Life Sciences and Medicine, Key Laboratory of Plant Secondary Metabolism and Regulation of Zhejiang Province, Zhejiang Sci-Tech University, Hangzhou, China; ^2^ Botany and Microbiology Department, Faculty of Science, Menoufia University, Shebin Elkoom, Egypt; ^3^ College of Plant Protection, China Agricultural University, Beijing, China; ^4^ Laboratory of Ornamental Plants, Department of Landscape Architecture, Zhejiang Sci-Tech University, Hangzhou, China; ^5^ Shaoxing Biomedical Research Institute of Zhejiang Sci-Tech University Co., Ltd, Zhejiang Engineering Research Center for the Development Technology of Medicinal and Edible Homologous Health Food, Shaoxing, China

**Keywords:** *S. miltiorrhiza*, tanshinones, MAPK, fungal elicitors, phylogenetic analysis

## Abstract

*Salvia miltiorrhiza* is a well-known traditional Chinese medicine (TCM) for its bioactive tanshinones that are used to treat various diseases and have high antimicrobial properties. Previous studies have shown that tanshinone accumulation in *S. miltiorrhiza* was shown to be significantly induced by fungal elicitors. Mitogen-activated protein kinases (MAPKs), which play critical roles in plant–microbe interactions and cellular processes, are known to regulate the accumulation of antimicrobial metabolites. In this study, we aimed to identify MAPK families in *S. miltiorrhiza* and screen SmMAPKs for candidates involved in fungal elicitor-mediated tanshinone accumulation. Through genome-wide analysis, we identified 17 MAPK, 7 MAPKK, and 22 MAPKKK genes in *S. miltiorrhiza*, which were distributed across nine chromosomes. Phylogenetic analysis classified SmMAPKs into two subgroups, TEY and TDY, similar to *Arabidopsis* MAPKs, while all SmMAPKKKs clustered under the MEKK subfamily. Cis-acting element analysis revealed that most SmMAPK genes are associated with stress and phytohormone responses suggesting their involvement in defense mechanisms. To investigate the role of MAPK s in tanshinone accumulation, hairy roots of *S. miltiorrhiza* were treated with two fungal elicitors, yeast extract and *Aspergillus niger*, for 1 and 4 days. HPLC analysis demonstrated that both elicitors significantly promoted the accumulation of tanshinones, particularly cryptotanshinone and dihydrotanshinone. Comprehensive transcriptomic analysis, followed by Pearson correlation coefficient analysis, revealed a strong positive correlation between tanshinone content and *SmMPK4* and *SmMPKK5*, while negative correlations were observed with *SmMPKKK6*, *SmMPKKK11*, and *SmMPKKK20*. The presence of defense-related cis-acting elements in the promoter regions of *SmMPK4*, *SmMPKK5*, *SmMPKKK6*, *SmMPKKK11*, and *SmMPKKK20* further supports their involvement in fungal elicitor-mediated tanshinone accumulation. This study provides critical insights into the regulatory roles of SmMAPK genes in tanshinone accumulation in *S. miltiorrhiza* in response to fungal elicitors. These findings have potential applications in enhancing tanshinone production for medicinal purposes offering a foundation for further research into the molecular mechanisms underlying tanshinone biosynthesis.

## Introduction

*Salvia miltiorrhiza* is a well-known traditional Chinese medicine (TCM) valued for its production of tanshinones, the primary bioactive compounds (Xia et al., 2023; Huang et al., 2024). These diterpenoids are clinically used to treat hepatocirrhosis, cardiovascular diseases (Ren et al., 2019), and menstrual pain (Lee et al., 2020). Beyond their therapeutic applications, tanshinones exhibit antimicrobial properties, with cryptotanshinone and dihydrotanshinone I demonstrating stronger activity than tanshinone I and tanshinone IIA (Zhao et al., 2011), suggesting their role as defensive metabolites. Notably, fungal elicitors significantly enhance tanshinone accumulation in *S. miltiorrhiza* (Ming et al., 2013; Contreras et al., 2019) further supporting their involvement in plant defense. Transcriptomic studies reveal that fungal induction upregulates genes in the tanshinone biosynthetic pathway (Zhou et al., 2017; Wu et al., 2022), though the precise regulatory mechanisms remain unclear. Given their dual antimicrobial and pharmacological potential, elucidating the biosynthetic regulation of tanshinones could enable biotechnological strategies to optimize their production for medical and agricultural applications. Fungal elicitation starts with a pathogen-associated molecular pattern (PAMP) that binds to the plant cell membrane through recognition receptors. The binding of PAMPs and membrane receptors activates the mitogen-activated protein kinase (MAPK) signaling pathway that results in the alleviation of transcription factor (TF) expression, which, in turn, activates the defensive metabolites biosynthesis genes (Zhai et al., 2017). For example, defensive metabolites biosynthesis genes were activated by different types of transcription factors in corn (Ibraheem et al., 2015), cotton (Xu et al., 2004), and rice (Yamamura et al., 2015).

Mitogen-activated protein kinases (MAPKs) are protein kinases that are involved in signaling pathways of various cellular processes, such as growth and development, metabolism, cell death, and defense responses (Zhang and Liu, 2002; Roux and Blenis, 2004; Wang et al., 2007; Widmann et al., 1999; Khan et al., 2024). In addition, they play a crucial role in plant–microbe interactions and trigger immune responses (Arthur and Ley, 2013; Guo et al., 2021; Van Gerrewey and Chung, 2024). In addition to their role in stress responses, MAPK cascades have been shown to regulate the biosynthesis of secondary metabolites in various plant species, including camalexin in *Arabidopsis* and momilactones in rice (Ren et al., 2008; Kishi-Kaboshi et al., 2010a; Kishi-Kaboshi et al., 2010b; Gaur et al., 2018; Li et al., 2024; Zhou et al., 2025). These findings highlight the potential of MAPK cascades as key regulators of defensive metabolite biosynthesis in plants. However, MAPKs have not been identified or characterized before in *S. miltiorrhiza*, and their role in fungal elicitor-mediated tanshinone accumulation has not been investigated yet.

In this study, we detected and comprehensively analyzed the SmMAPK gene families in *S. miltiorrhiza*. SmMAPK gene chromosomal distribution, phylogenetic relationships, protein alignments, conserved motifs, conserved domains, cis-acting elements, and gene structure were investigated. We treated hairy roots of *S. miltiorrhiza* with two fungal elicitors, yeast extract and *Aspergillus niger*, that significantly promoted tanshinone accumulation. Then, we employed Pearson correlation coefficient analysis to screen SmMAPK genes that may have a potential role in tanshinone accumulation in *S. miltiorrhiza*. This study provides new insights into revealing the potential roles of SmMAPK genes in regulating the fungal elicitor-mediated tanshinone accumulation in *S. miltiorrhiza.*

## Material and methods

### Plant materials and treatments

The hairy roots of *S. miltiorrhiza* were cultured at Zhejiang Sci-Tech University, Hangzhou, China. Approximately 0.2 g of hairy roots with the same chronological age (21 days post-induction) and active growth status (visible root tip elongation) to ensure biological consistency across replicates were inoculated into 50 ml of MS 6,7-V liquid culture medium. The hairy roots were cultured in a shaking incubator at 110 rpm/min. Three-week-old hairy roots were treated with 100 mg/L of yeast extract and *A. niger* elicitors. After 1, 4, and 7 days, hairy roots were collected for HPLC and RNA sequencing. Immediately after harvest, hairy root samples were flash-frozen in liquid nitrogen, stored at −80°C in RNase-free tubes, and transferred to dry ice during transport to prevent thawing. Each group had three biological replicates.

### Preparation of elicitors

*Aspergillus niger* ATCC 6275 freeze-dried powder was purchased from Hunan Fenghui Biotechnology Co., Ltd. and yeast extract powder (LP0021B) from Oxoid. The *Aspergillus niger* freeze-dried powder was activated; then, the elicitor was prepared using the acid hydrolysis method of Wen-Zhi et al. (2002), while we prepared the yeast extract elicitor following the method of Shi et al. (2014). Both elicitors’ mass concentrations were expressed as sugar mass concentrations. The sugar concentration was determined using the anthrone colorimetric (micro-method) soluble sugar content determination kit. The glucose standard curve drawn by the glucose content (x) against the absorbance (y) in the kit is y = 4.275x − 0.07, where x and y represent standard concentration (mg/ml) and absorbance value, respectively.

### Extraction and HPLC detection of tanshinones from hairy roots

Air-dried hairy roots for 4 days at 40°C were ground into powder using a sample grinder (Osheng Instrument, Bioprep-24). Into a centrifuge tube, 0.02 g was weighed using an analytical balance, and 1 ml of 70% methanol (Macklin, M813907-4L) solution was added. The tube was placed in an ultrasonic extractor for ultrasonic extraction for 1 h. The sample was then centrifuged at 12,000 rpm for 15 min, and the supernatant was filtered using a 0.22-μm oil filter (Biosharp, BS-QT-013). The filtrate was collected in a sample injection bottle. A phosphoric acid water with 0.02% concentration was prepared as the mobile phase, using ultrapure water and phosphoric acid (Macklin, P816338—500 ml) after filtration, and then ultrasonic degassing was performed. Secondary metabolites were detected by high-performance liquid chromatography under the following: high-performance liquid chromatography (Waters 2695) and diode array detector (Waters 2998) were used for detection, the chromatographic column was Waters SunFire C18 column, chromatographic acquisition and analysis were completed by Empower 2 software, and the loading volume was 20 µl. The chromatographic conditions used were a flow rate of 1 ml/min and a column temperature of 25°C. The detection wavelengths were 270 nm. External standard analyte peak area was used to determine the tanshinone compound content. Three replicates were used for all samples to ensure accuracy.

### Extraction and sequencing of RNA

TRIzol^®^ Reagent was used for RNA extraction. RNA integrity was evaluated with the 5300 Bioanalyzer (Agilent), and quantification was performed using the ND-2000 spectrophotometer (NanoDrop Technologies). Only high-quality RNA sample (OD260/280 = 1.8–2.2, OD260/230 ≥ 2.0, RIN ≥ 6.5, 28S:18S ≥ 1.0, > 1 μg) was used to construct a sequencing library.

RNA sequencing was done by Shanghai Majorbio Biopharm Biotechnology Co., Ltd., China, per the manufacturer’s guidelines (Illumina, San Diego, CA). RNA-seq libraries were prepared using the Illumina^®^ Stranded mRNA Prep, Ligation protocol. Shortly, messenger RNA was isolated according to the polyA selection method by oligo(dT) beads and then fragmented by fragmentation buffer first. Second, double-stranded cDNA was synthesized using a SuperScript double-stranded cDNA synthesis kit (Invitrogen, CA) with random hexamer primers (Illumina). Then, the synthesized cDNA was subjected to end repair, phosphorylation, and “A” base addition according to Illumina’s library construction protocol. Libraries were size selected for cDNA target fragments of 300 bp on 2% Low Range Ultra Agarose followed by PCR amplified using Phusion DNA polymerase (NEB) for 15 PCR cycles. After quantification by Qubit 4.0, the paired-end RNA-seq sequencing library was sequenced with the NovaSeq 6000 sequencer (2 × 150-bp read length).

### Identification and characterization of MAPK gene family in *S. miltiorrhiza*

The gene sequences of MAPKs of *Arabidopsis thaliana* (20 AtMAPKs, 10 AtMAPKKs, and 80 AtMAPKKKs) were downloaded from the *A. thaliana* database TAIR (Berardini et al., 2015), *Oryza sativa* (17 OsMAPKs, 8 OsMAPKKs, and 75 OsMAPKKKs) from the Rice Genome Annotation Project database (Kawahara et al., 2013), cucumber (14 CsMAPK, 6 CsMAPKK, and 59 CsMAPKKK) from the Cucumber Genomics Database, and the *S. miltiorrhiza* genome data were retrieved from the GWH database (Pan et al., 2023).

Nucleotide BLAST was carried out with a cutoff of E-value <1 × 10^−5^ as the query and search for the MAPK genes within the *S. miltiorrhiza* genome. HMM profiles of the protein kinase domain (PF00069) were sourced from the Pfam database, and the NCBI database (Wang et al., 2023) was used to confirm the presence of the conserved domain within the candidate MAPK.

The theoretical isoelectric point (pI) and molecular weight (MW) of the MAPK genes were determined using the Expasy online tool (Gasteiger et al., 2005). The subcellular localization of MAPK proteins was predicted via the BUSCA online platform (Savojardo et al., 2018). The chromosomal localization of MAPK genes was analyzed by the TBtools software (Chen et al., 2023).

### Synteny analysis

To explore the evolutionary conservation of MAPK genes, we performed synteny analysis by comparing the genomic locations of MAPK genes in *S. miltiorrhiza* with those in closely related species such as *Arabidopsis thaliana* and *Oryza sativa*. We used MCScanX to identify orthologous genes and analyzed gene order conservation across species. Tbtools was used to visualize the synteny analysis maps.

### Phylogenetic analysis, amino acid sequences alignments, conserved motifs, domain analyses, cis-acting regulatory elements, and gene structure analysis

Phylogenetic trees were generated using MEGA 11 software with the neighbor-joining (NJ) method, and a bootstrap analysis was conducted with 1,000 iterations. Phylogenetic trees were visualized using the iTOL v6 online tool (https://itol.embl.de/). Amino acid sequence alignments were done by MUSCLE, and GENEDOC software was used to visualize the alignments and detect the conserved motif signatures. Ten motifs were identified from SmMAPK proteins using the MEME online website (https://meme-suite.org/meme/tools/meme). Conserved domain analysis was done by the NCBI database (https://www.ncbi.nlm.nih.gov/). The cis-acting element analysis was done by PlantCARE online. Conserved domains, distribution of cis-acting elements in the promoters, and SmMAPK gene structure were visualized using TBtools software.

### Protein–protein interaction network and functional annotation analysis

Protein–protein interaction (PPI) networks were constructed using the STRING database. Interactions were selected based on a confidence score threshold of 0.4. Gene Ontology (GO) enrichment analysis and KEGG pathway analysis were performed and visualized using the STRING database. Enrichment was evaluated using default parameters.

### Differential expression analysis and Pearson correction analysis

Gene expression analysis was performed using RNA-seq data. Differential expression was determined with DESeq2 using thresholds of |log2FC| ≥ 0.5 and FDR < 0.05. Heatmaps visualizing normalized expression of differentially expressed genes (DEGs) were generated using TBtools. All fold change, log2 fold change values, regulation, and significance are summarized in **Supplementary Table S5**. For co-expression analysis, pairwise Pearson correlations were computed from normalized expression values and plotted as a correlation matrix using the SRplot online platform. Correlations with a p-value < 0.05 were considered statistically significant.

### Gene expression validation by RT-qPCR

Real-time quantitative polymerase chain reaction was used to analyze the relative expression levels of SmMAPKs candidate genes. SYBR^®^ Green Pro Taq HS premixed qPCR kit (AG, AG11701) was used for fluorescence quantitative PCR reaction. QuantStudio 6 Flex Real-Time PCR System (ThermoFisher, USA) was used to analyze the expression of SmMAPKs genes. Actin was chosen as the internal control for normalization in qPCR, and three biological replicates were used. The experiments were performed with the following operating parameters: sample pass initial denaturation at 95°C for 30 s, then pass 40 amplification cycles. Each cycle consisted of denaturation at 95°C for 5 s and annealing at 60°C for 30 s. The last stage of dissociation was 95°C for 15 s, 65°C for 1 min, and 95°C for 15 s. Primers used for RT-qPCR are listed in **Supplementary Table S6**.

### Statistical analysis

Data analysis and ANOVA tests were done by Prism 9.0.0. Error bars represent the mean value of standard deviation ± for three independent replicas. One-way ANOVA multiple comparison test was used to determine significant differences. (****) represents a significant difference at the level of p < 0.0001 relative to the control group.

## Results

### Genome-wide identification and chromosomal mapping of MAPK genes in *S. miltiorrhiza*

**Figure 1:**
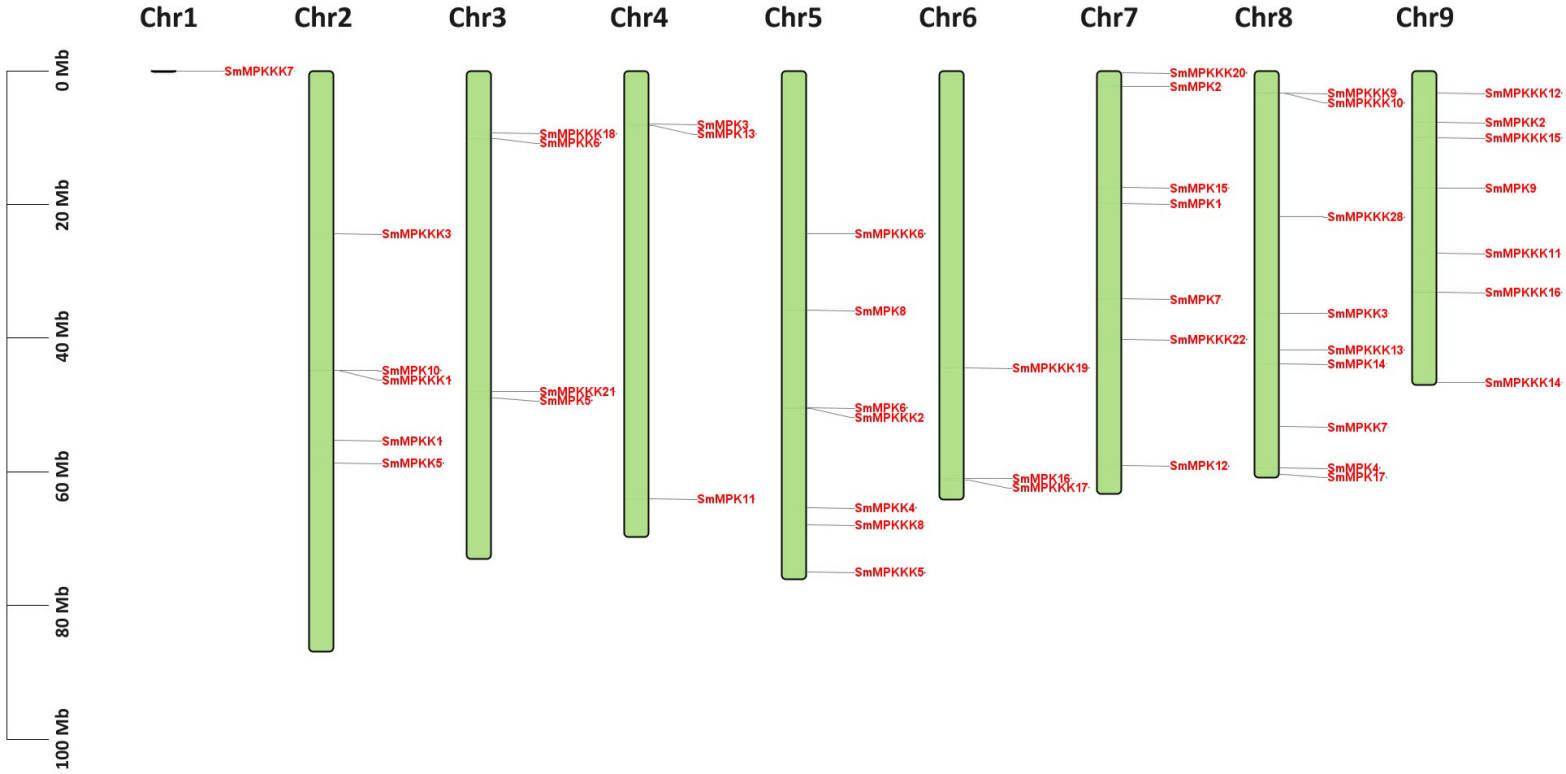
Chromosome mapping of the *S. miltiorrhiza* MAPK gene families. The scale on the left represents the chromosome lengths. The green boxes represent the chromosomes. MAPK gene members are marked in red.

A total of 17 MAPK, 7 MAPKK, and 22 MAPKKK genes were identified from *S. miltiorrhiza* genome (**Supplementary Table S1**). The sequence of cDNA and proteins of identified MAPK genes are listed in **Supplementary Table S2** and **Supplementary Table S3**, respectively. The cDNA length of identified MAPK genes ranged from 708 bp in *SmMPKKK20* to 2,877 bp in *SmMPKKK2*. The predicted MW of the MAPK proteins ranged from 25,860.58 Da in *SmMPKKK20* to 95,481.2 Da in *SmMPKKK21*, while their theoretical pI ranged from 4.51 in *SmMPKKK6* to 9.52 in *SmMPKKK21*. Subcellular localization analysis revealed that 32 MAPK proteins are localized in the nucleus, 7 MAPK proteins were predicted to localize in the chloroplast, 6 proteins are located in the cytoplasm, while only *SmMPKKK22* is located in the endomembrane system. Chromosome mapping revealed that *S. miltiorrhiza* MAPK genes are located on nine chromosomes (**Figure 1**). Chromosome 8 has the biggest number of MAPK genes as it has nine genes, while only one gene (*SmMPKKK7*) is located on chromosome 1. Chromosomes 5, 7, and 9 all have the same number of MAPK genes (seven) while chromosome 2 has five genes, and both chromosomes 3 and 5 have three genes.

### Synteny analysis

**Figure 2:**
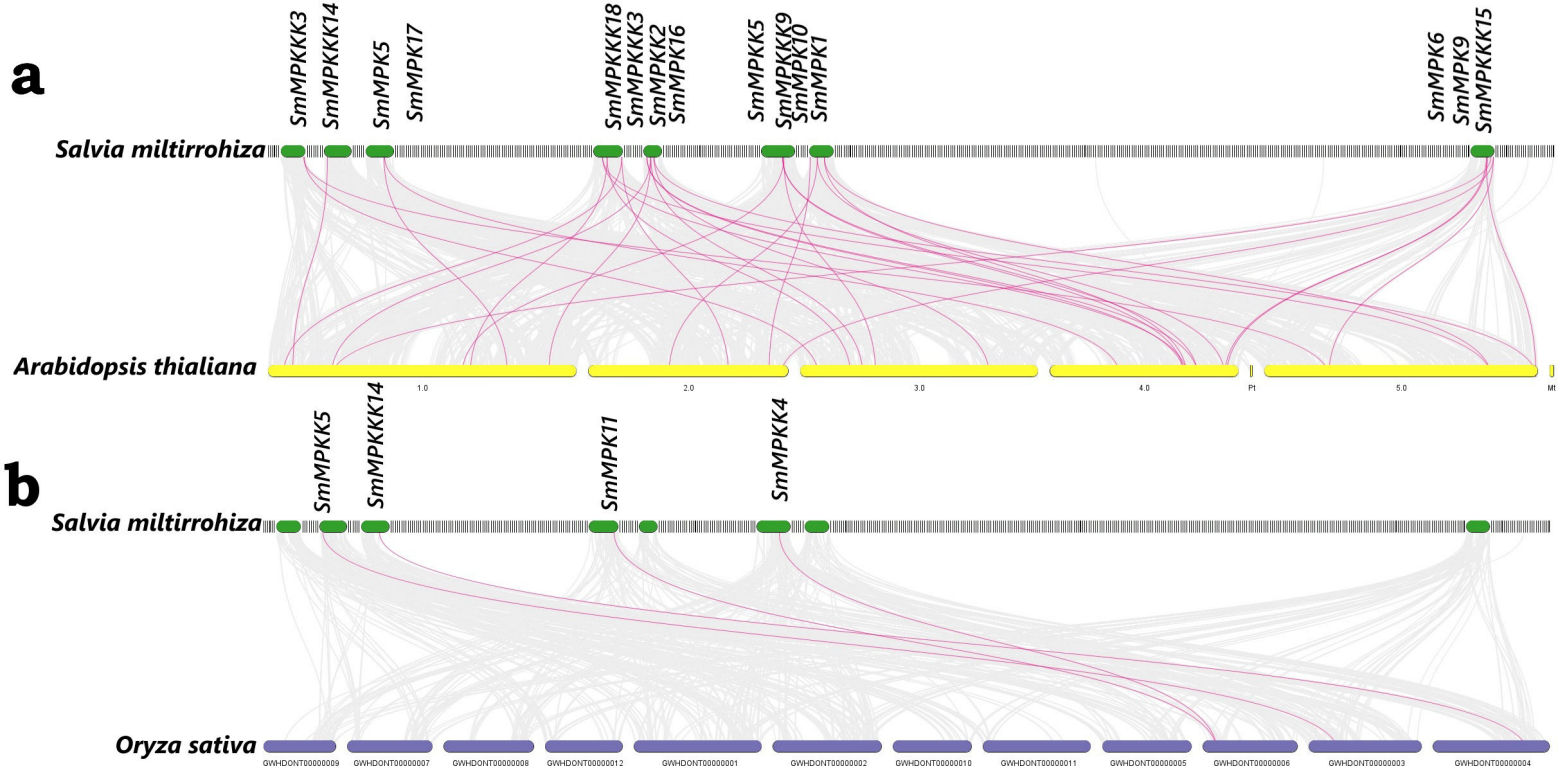
Synteny analysis of MAPK genes between **(A)** *S. miltiorrhiza* and *A. thaliana*; **(B)** *S. miltiorrhiza* and *O. sativa*. The red lines represent the syntenic MAPK gene pairs, while the gray lines represent the syntenic blocks within the genomes.

Our synteny analysis (**Figure 2**) revealed that 21 MAPK genes in *S. miltiorrhiza* exhibit conservation with orthologs in *A. thaliana* and 4 genes in *O. sativa*. This suggests that the WAK/WAKL family has been preserved throughout evolution.

### Phylogenetic analysis of MAPK genes

**Figure 3:**
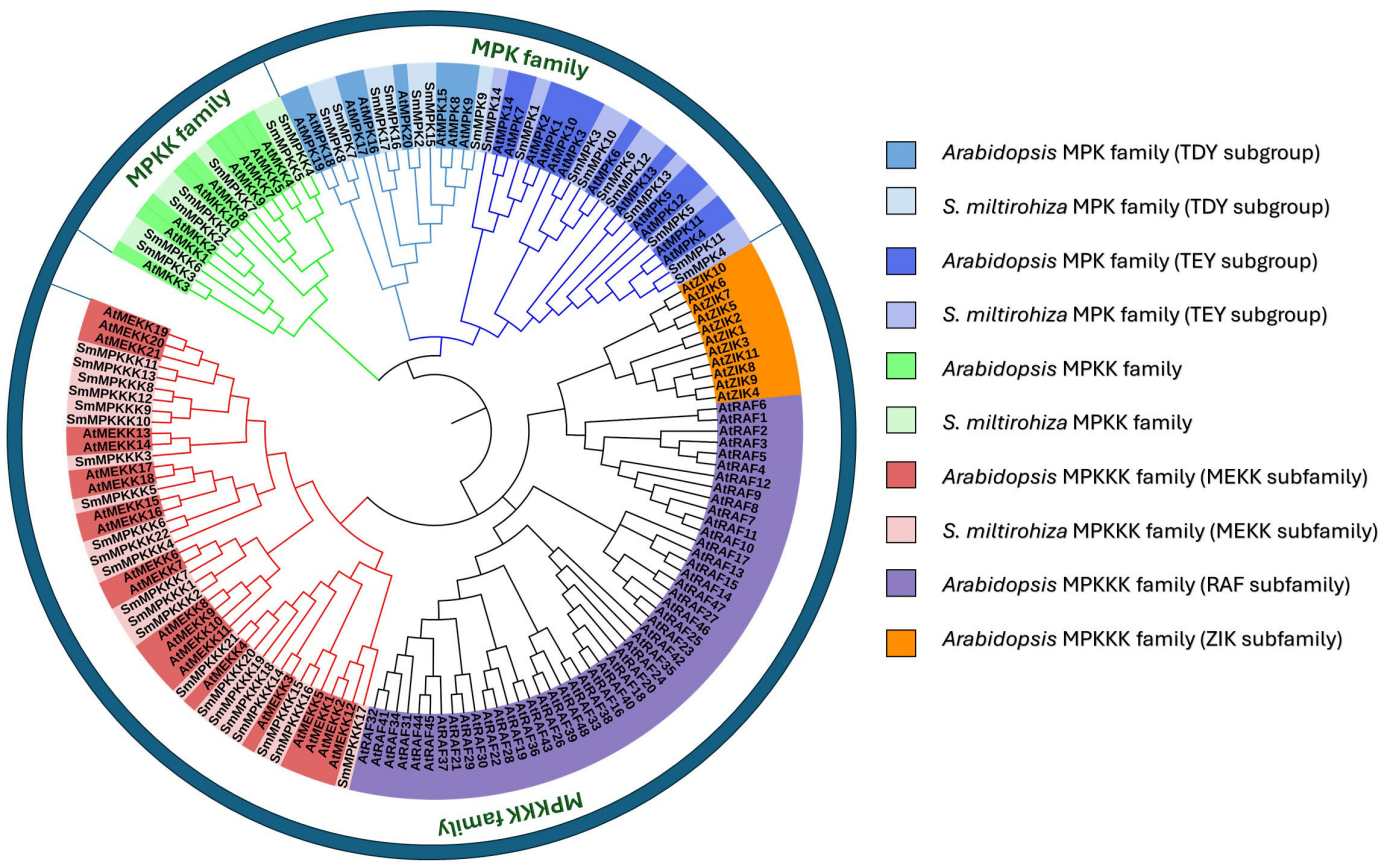
Phylogenetic tree of *S. miltiorrhiza* and *A. thaliana* MAPK gene families.

To investigate the evolutionary relationships of the identified MAPK genes, a phylogenetic tree was generated (17 MAPKs, 7 MAPKKs, and 22 MAPKKKs) from *S. miltiorrhiza*, together with 20 MAPKS, 10 MAPKKS, and 80 MAPKKKS from *Arabidopsis thaliana*, using the neighbor-joining (NJ) method. The protein sequences of *Arabidopsis* MAPK are listed in **Supplementary Table S3**. The MAPK proteins of each subfamily from the two species were grouped together in a separate group. Moreover, most of the MAPK proteins from *S. miltiorrhiza* clustered with their homologs in *A. thaliana*. Interestingly, all proteins from the family MAPKKKS clustered with *A. thaliana* MEKK subfamily (**Figure 3**).

### MAPK characterization

**Figure 4:**
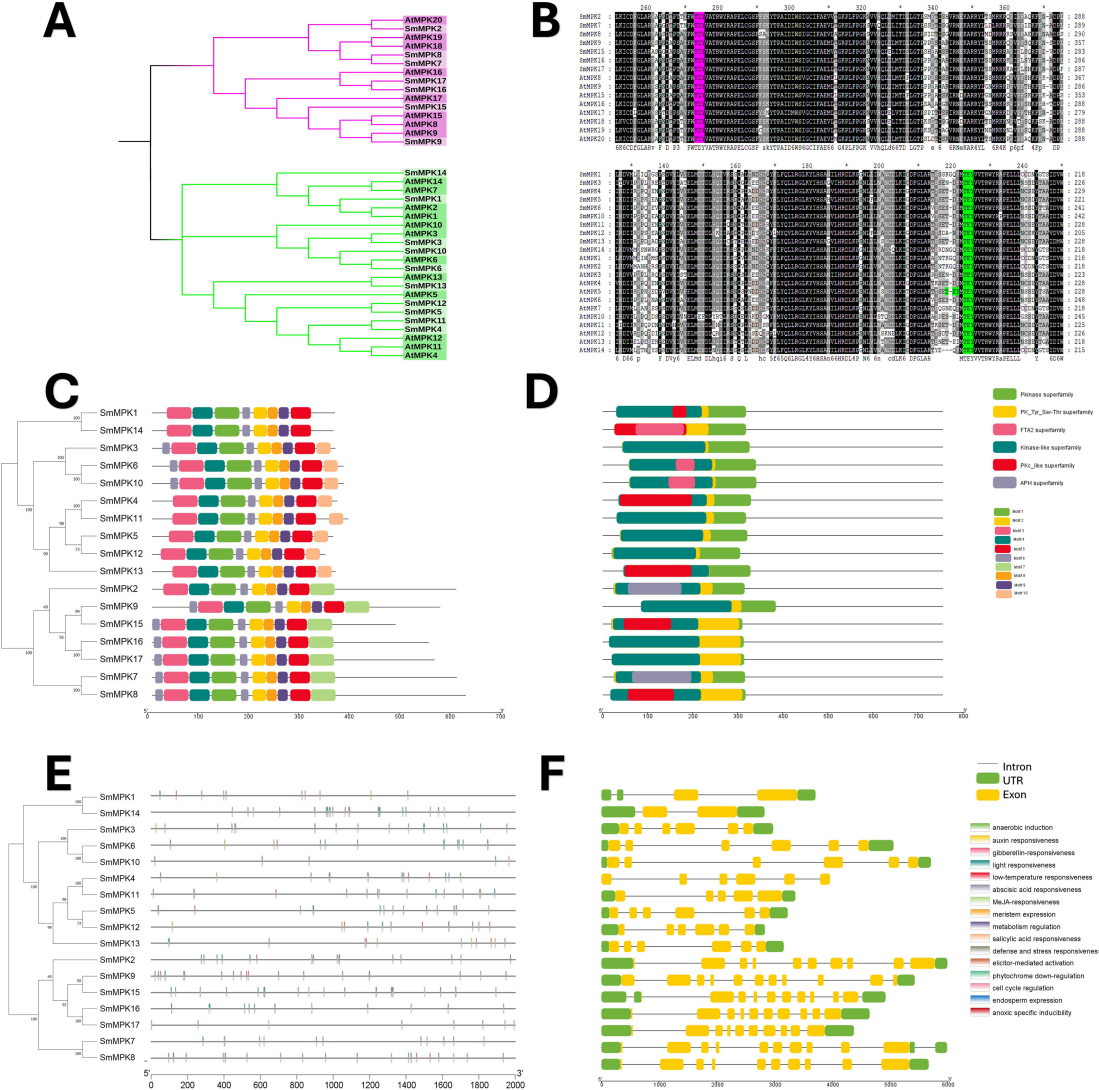
characterization analyses of *S. miltiorrhiza* MAPK gene family. **(A)** Phylogenetic tree of *S. miltiorrhiza* and *A. thaliana* MAPK proteins. The phylogenetic tree was done by the neighbor-joining (NJ) method with a 1,000-iteration bootstrap. TDY group with pink color and TEY group in green. **(B)** Alignments of *S. miltiorrhiza* and *A. thaliana* MAPK amino acid sequences. Conserved signatures are highlighted by different colors. **(C)** Conservative motif distribution of SmMAPK proteins. Boxes with different colors represent the 10 different motifs identified by meme suit. **(D)** Conserved domains of SmMAPK gene family. Boxes with different colors represent the different conserved domains identified. **(E)** Cis-elements in promoters of SmMAPK genes. Cis-acting elements with different functions are represented by different colored boxes. **(F)** The gene structure of SmMAPKs. Black lines represent introns, green boxes represent untranslated regions, and yellow boxes represent exons.

The *Salvia miltiorrhiza* MAPK (SmMAPK) gene family was characterized through comparative analysis with *Arabidopsis thaliana*. Phylogenetic tree construction (**Figure 4A**) revealed that all 17 SmMAPK proteins cluster into the two conserved *Arabidopsis* subgroups defined by their TxY phosphorylation motifs (TEY or TDY) (Ichimura et al., 2002b). Sequence alignments confirmed these motifs in SmMAPKs (**Figure 4B**). MEME analysis identified 10 conserved motifs (**Figure 4C**; **Supplementary Table S4**), with motifs 2, 5, 8, and 9 forming the Pkinase domain. Motif 7 was exclusive to the TDY-subtype C-termini, while motif 10 predominated in the TEY-subtype members. Domain analysis (**Figure 4D**) showed that all SmMAPKs contain Pkinase and PK-Tyr-Ser-Thr domains, with FTA2 (TEY subgroup) and APH (TDY subgroup) domains restricted to specific members. Promoter analysis of 2-kb upstream regions (**Figure 4E**) identified 164 stress-responsive elements, including 5 defense-related elements in *SmMPK4/7/17* (with *SmMPK4* harboring an elicitor-responsive element). Hormone-responsive elements were abundant (78 total), particularly for MeJA (26) and ABA (29). Only 24 growth-related elements were detected, which were absent in 5 *SmMAPKs*. Gene structure analysis (**Figure 4F**) showed that TDY-subtype genes uniformly contain 10 exons, while TEY-subtype genes typically have 6 (except *SmMPK1/14* with 2). These results suggest that SmMAPKs primarily regulate stress and hormone responses, with structural divergence between subgroups.

**Figure 5:**
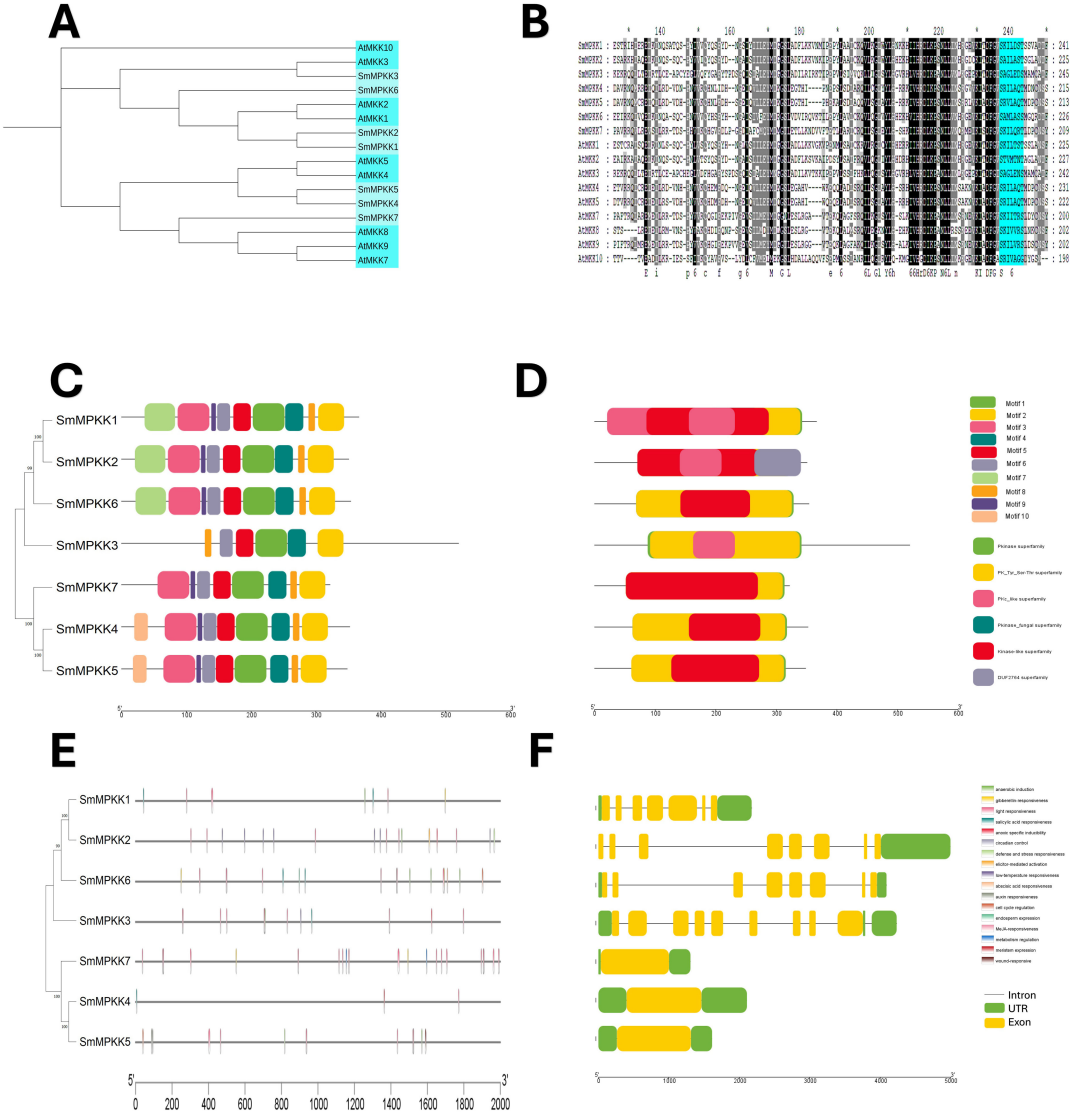
Characterization analyses of *S. miltiorrhiza* MAPKK gene family. **(A)** Phylogenetic analysis of *S. miltiorrhiza* and *A. thaliana* MAPKK proteins. The phylogenetic tree was done by the neighbor-joining (NJ) method with a 1,000-iteration bootstrap. **(B)** Alignments of amino acid sequences of *S. miltiorrhiza* and *A. thaliana* MAPKK gene family. Conserved signatures are highlighted by different colors. **(C)** Conservative motif distribution of SmMAPKK proteins. Boxes with different colors represent the 10 different motifs identified by meme suit. **(D)** Conserved domains of SmMAPKK gene family. Boxes with different colors represent the different conserved domains identified. **(E)** Cis-elements in promoters of SmMAPKK genes. Cis-acting elements with different functions are represented by different colored boxes. **(F)** The gene structure of SmMAPKKs. Black lines represent introns, green boxes represent untranslated regions, and yellow boxes indicate exons.

A neighbor-joining phylogenetic tree of *Arabidopsis thaliana* (10) and *Salvia miltiorrhiza* (7) MAPKK proteins revealed close evolutionary relationships, with all SmMAPKKs nested within *Arabidopsis* clades (**Figure 5A**). Sequence alignments confirmed the conserved MAPKK motif [S/T]xxxxx[S/T] in all seven SmMAPKKs (**Figure 5B**). MEME analysis identified 10 motifs (**Figure 5C**; **Supplementary Table S4**), with subgroup-specific distributions: Group I (SmMAPKK1/2/6) contained N-terminal motif 7 but lacked motif 10, Groups II (SmMAPKK3) and III (SmMAPKK7) lacked both motifs, and Group IV (SmMAPKK4/5) exclusively contained motif 10. Domain analysis showed that all members harbor Pkinase and PK-Tyr-Ser-Thr domains, except SmMAPKK2, which uniquely possessed a DUF2764 domain (**Figure 5D**). Promoter analysis detected 61 stress-responsive elements, including defense-related elements in *SmMAPKK2* (elicitor responsive) and *SmMAPKK5*, and 32 hormone-responsive elements (MeJA: 6; ABA: 12). Notably, *SmMAPKK2* lacked hormone-responsive elements. Gene structure analysis divided SmMAPKKs into two subgroups as follows: one with single-exon genes (*SmMAPKK4/5/7*) and another with multi-exon genes (*SmMAPKK1/2/3/6*) containing seven to eight exons (**Figure 5F**). These findings suggest functional diversification among SmMAPKK subgroups in stress and hormonal responses.

**Figure 6:**
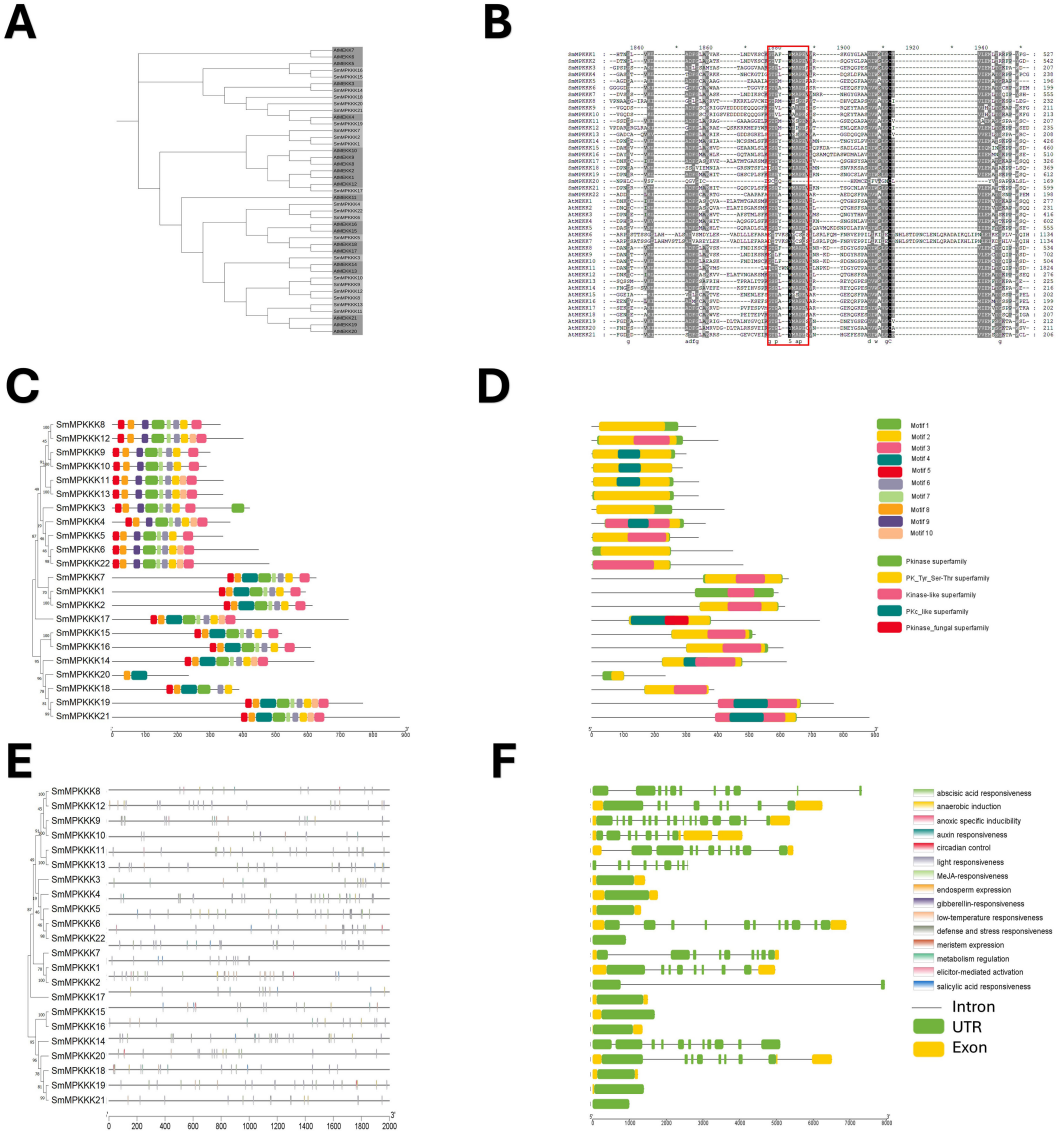
Characterization of *S. miltiorrhiza* MAPKKK gene family. **(A)** Phylogenetic tree of *S. miltiorrhiza* and *A. thaliana* MAPKKK proteins was generated using the neighbor-joining (NJ) method, and a bootstrap analysis was conducted with 1,000 iterations. **(B)** Alignments of amino acid sequences of *S. miltiorrhiza* and *A. thaliana* MAPKKK gene family. Conserved signatures are highlighted by different colors. **(C)** Conservative motif distribution of SmMAPKKK proteins. Boxes with different colors represent the 10 different motifs identified by meme suit. **(D)** Conserved domains of SmMAPKKK gene family. Boxes with different colors represent the different conserved domains identified. **(E)** Cis-elements in promoters of SmMAPKKK genes. Cis-acting elements with different functions are represented by different colored boxes. **(F)** The gene structure of SmMAPKKKs. Black lines represent introns, and green and yellow boxes represent untranslated regions and exons, respectively.

Phylogenetic analysis revealed close evolutionary relationships between *S. miltiorrhiza* MAPKKKs and *A. thaliana* MEKK subfamily (**Figures 6A, B**). The conserved MEKK motif G[T/S]Px[W/Y/F]MAPEV was present in all SmMAPKKKs except SmMAPKKK20. MEME analysis identified 10 motifs (**Figure 6C**; **Supplementary Table S4**), with subgroup-specific distributions as follows: Group I contained motif 9 (absent motif 4), while Groups II–IV retained motif 4 but lacked motif 9. All members harbored Pkinase and PK-Tyr-Ser-Thr domains (**Figure 6D**). Promoter analysis detected 261 stress-responsive elements (primarily light/low-temperature related), with 17 defense elements across 12 genes, including an elicitor-responsive element in *SmMPKKK11*. Hormone-responsive elements (196 total) were abundant, dominated by MeJA (92) and ABA (62) elements. Gene structure varied widely ranging from intronless (*SmMPKKK21/14/2/13/8*) to four-exon (*SmMPKKK10*) architectures (**Figure 6F**). These findings highlight SmMAPKKKs’ roles in stress and hormonal signaling.

### Protein-protein interaction network analysis

**Figure 7:**
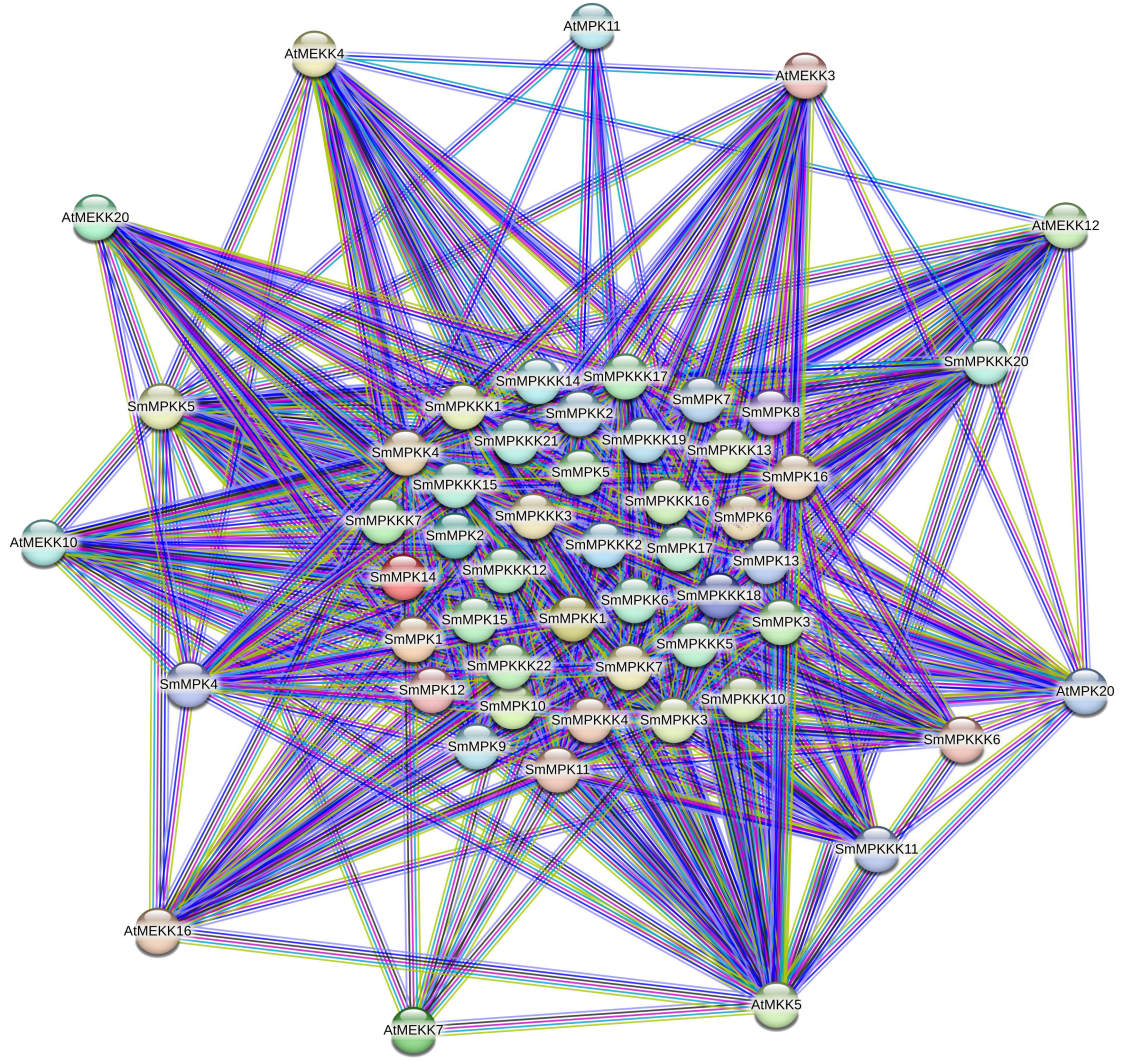
Protein–protein interaction (PPI) network illustrating interactions between the identified SmMAPKs proteins and their orthologs of *Arabidopsis*. Nodes represent proteins, and edges indicate known and predicted interactions. The thickness of the edges corresponds to the confidence score of the interaction. Different edge colors represent different interactions such as experimental data, co-expression, databases, or text mining.

To gain insights into the functional relationships and potential signaling pathways involving the identified MAPK genes in *S. miltiorrhiza*, a protein–protein interaction (PPI) network was constructed using the *A. thaliana* MAPK orthologs as a reference. The PPI network (**Figure 7**) revealed significant interactions between *S. miltiorrhiza* MAPKs and their *Arabidopsis* counterparts highlighting conserved regulatory mechanisms across species.

Our results show that SmMPKK5 strongly interacts with AtMEKK10 (combined score: 0.854), an MAPKKK involved in ABA signaling and drought stress responses (Danquah et al., 2015; Xing et al., 2008). This suggests that SmMPKK5 may participate in ABA-mediated abiotic stress signaling in *S. miltiorrhiza*. Additionally, SmMPKKK6, SmMPKKK11, and SmMPKKK20 show interactions with AtMEKK12, AtMEKK16, and AtMEKK20, which are known regulators of PAMP-triggered immunity (PTI) and MAPK cascades responding to pathogen infection (Asai et al., 2002; Gao et al., 2008; Wang et al., 2017). For example, AtMEKK1 (closely related to AtMEKK12) initiates PTI signaling by activating downstream MAPKs like MPK3/6 in response to bacterial and fungal elicitors (Meng and Zhang, 2013). AtMEKK16 has also been associated with stress signal integration, including responses to wounding and oxidative stress (Ichimura et al., 2002a). These interactions suggest that the *S. miltiorrhiza* MAPKs may act within conserved stress signaling pathways regulating responses to both abiotic stresses (e.g., salt, drought) and biotic stimuli (e.g., fungal elicitors).

### Gene Ontology enrichment analysis

**Figure 8:**
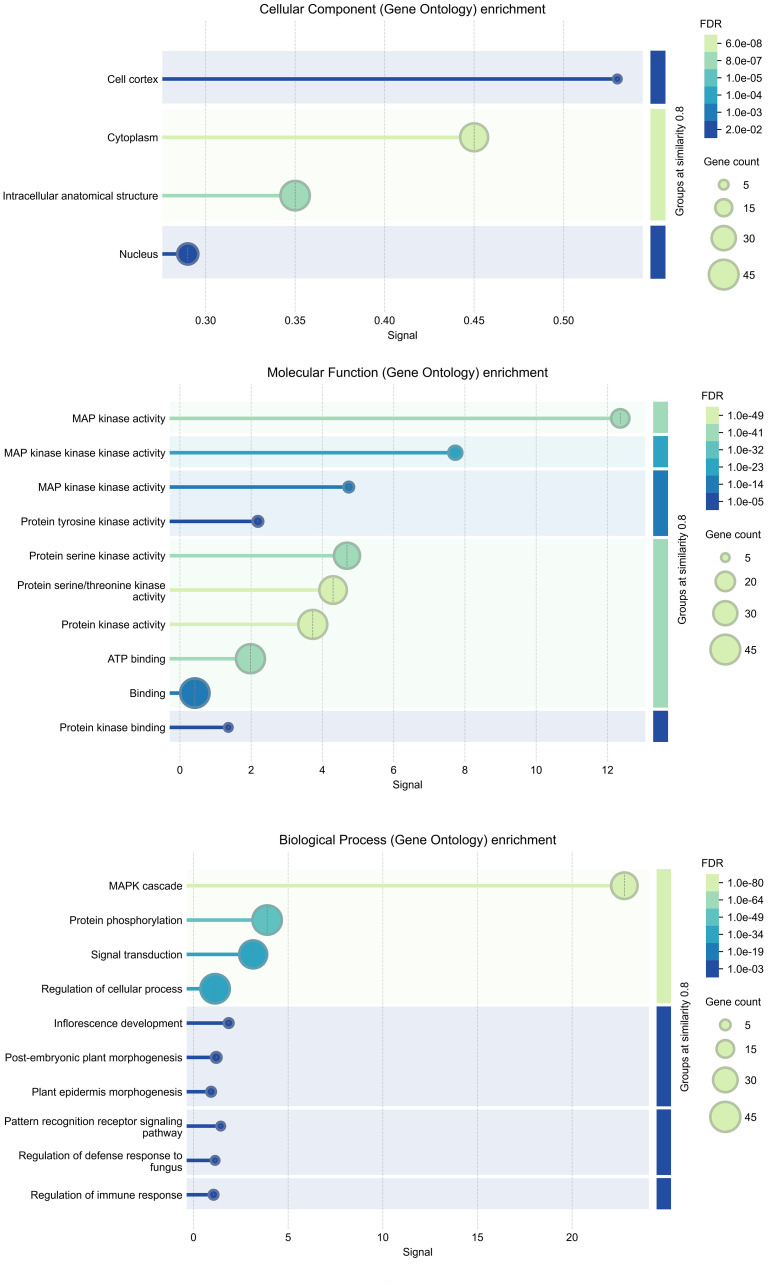
GO analysis of SmMAPKs genes. GO, Gene Ontology.

To investigate the functional roles of MAPK cascade genes in *Salvia miltiorrhiza*, GO enrichment analysis was conducted revealing significant associations across cellular components, molecular function, and biological process categories (**Figure 8**). Most MAPK genes were localized to the cytoplasm (40 genes) and nucleus (23 genes), indicating their involvement in both signal transduction and gene regulation. In the biological process category, 35 genes were directly associated with the MAPK cascade, 43 with protein phosphorylation and regulation of cellular processes, and 39 with signal transduction, underscoring the widespread involvement of MAPK genes in signaling and cellular regulation. Additionally, eight genes were linked to defense responses highlighting the potential roles of these kinases in plant immunity and stress adaptation. Molecular function enrichment showed a strong bias toward kinase-related activities, with 43 genes exhibiting protein kinase activity and ATP binding, and 18 specifically annotated with MAP kinase activity. Notably, *SmMPK4* displayed enrichment in MAP kinase activity, ATP binding, and nucleotide interactions consistent with its role as a core MAPK in stress-responsive signaling. *SmMPKK5* was significantly associated with MAPK kinase activity and phosphotransferase functions positioning it as a key MAPKK that likely activates downstream MAPKs such as *SmMPK4*. *SmMPKKK6* exhibited strong enrichment in protein kinase and kinase-binding functions suggesting its role in initiating MAPK cascades by interacting with and activating MAPKKs. *SmMPKKK11* showed general kinase activity and ATP binding implying a more basal regulatory function, while *SmMPKKK20* was distinctly enriched in MAPKKK activity indicating a specialized role in transducing upstream signals from membrane-based stimuli like pathogen recognition or hormonal cues. All GO annotation processes are summarized in **Supplementary Table S7**.

### KEGG pathway enrichment analysis

**Figure 9:**
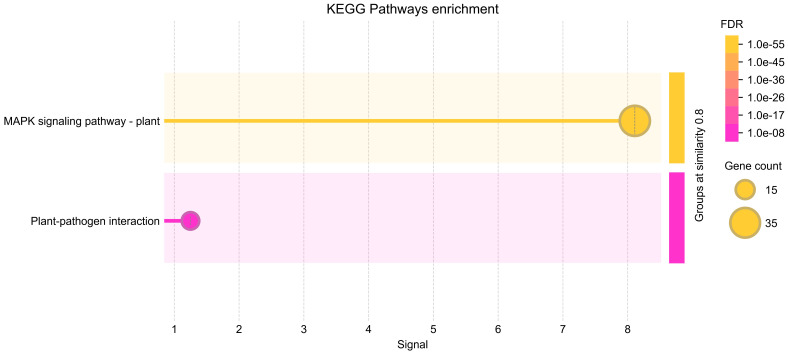
KEGG enrichment analysis of SmMAPKs genes. KEGG, Kyoto Encyclopedia of Genes and Genomes.

To further elucidate the functional roles of the identified MAPK genes in *Salvia miltiorrhiza*, KEGG pathway enrichment analysis was performed (**Figure 9**). The analysis revealed significant enrichment in pathways related to signal transduction and plant–pathogen interactions providing insights into the biological processes regulated by these genes. The most significantly enriched pathway was the MAPK signaling pathway—plant, with 36 genes associated with this pathway. This finding confirms the central role of the identified MAPK genes in MAPK signaling, which is known to regulate various cellular processes, including stress responses, growth, and development. The enrichment of these genes in the MAPK signaling pathway underscores their importance in transmitting extracellular signals to intracellular responses, particularly in the context of fungal elicitor-mediated tanshinone accumulation. Another significantly enriched pathway was plant–pathogen interaction, with 13 genes associated with this pathway. This pathway is crucial for plant defense mechanisms against pathogens, and the enrichment of MAPK genes in this pathway suggests their involvement in plant immunity and stress responses. The presence of these genes in the plant–pathogen interaction pathway highlights their potential role in mediating defense responses to fungal elicitors, which may, in turn, regulate tanshinone biosynthesis.

### Fungal elicitors promoted tanshinone accumulation in *S. miltiorrhiza*

**Figure 10:**
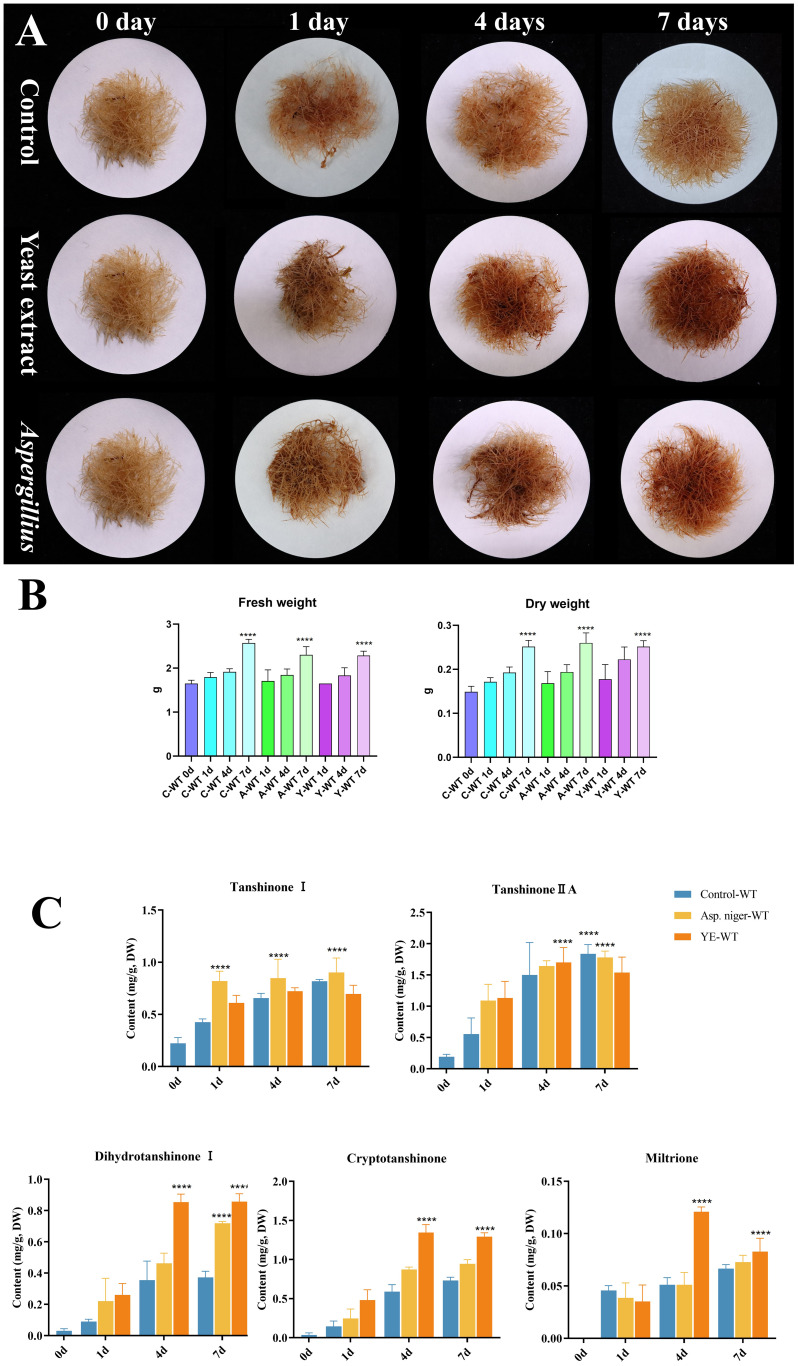
Effect of yeast extract and **(A)** *niger* elicitors on growth and tanshinone accumulation in *S. miltiorrhiza* hairy roots. **(A)** *S. miltiorrhiza* hairy roots treated by the two fungal elicitors for 1, 4, and 7 days. **(B)** Fresh and dry weight of *S. miltiorrhiza* hairy roots treated by the two fungal elicitors. **(C)** Tanshinone content in *S. miltiorrhiza* hairy roots treated by the two fungal elicitors. Error bars indicate the mean ± standard deviation (SD) based on three biological replicates. One-way ANOVA multiple comparison test was used to determine significant differences. (****) represents a significant difference at the level of p < 0.0001 relative to the control group (0-day treatment).

To investigate the relationship between MAPK gene families and tanshinone induction by fungal elicitation, hairy roots were treated by two fungal elicitors: yeast extract and *A. niger* (**Figure 10A**). The results indicate that both elicitors do not have a significant effect on the fresh and dry weight of hairy roots (**Figure 10B**). However, yeast extract and *A. niger* significantly promoted the accumulation of dihydrotanshinone, cryptotanshinone, and miltirone in *S. miltiorrhiza* hairy roots, but no significant change in tanshinone I and tanshinone IA was observed. Moreover, the effect of yeast extract elicitor on the accumulation of tanshinones was higher than that of *A. niger* (**Figure 10C**).

### Fungal elicitor-activated SmMAPK genes involved in tanshinone accumulation in *S. miltiorrhiza*

**Figure 11:**
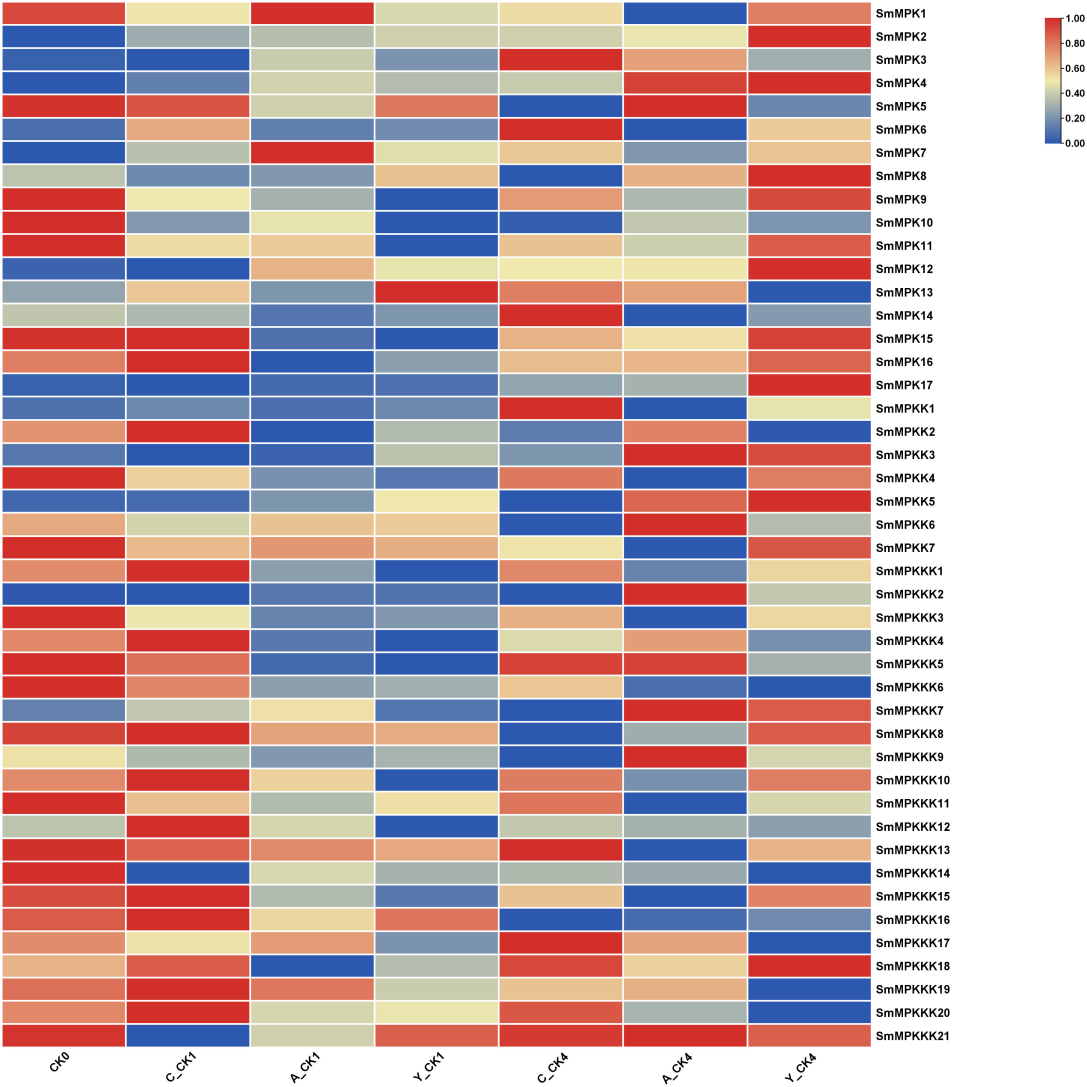
Heatmap of differentially expressed MAPK genes (DEGs) in fungal elicitor-treated hairy roots. Hierarchical clustering of normalized expression values (log2-transformed TPM/FPKM/variance-stabilized counts) for genes with significant differential expression (|log2FC| ≥ 0.5, FDR-adjusted p < 0.05) between treatment and control. Rows represent genes; columns represent samples grouped by condition. Expression levels are scaled by row and depicted as a color gradient (blue: downregulated; red: upregulated; yellow: mean expression). C_CK0, control group before treatment; C_CK1, control group 1 day after treatment; C_CK4, control group 4 days after treatment; A_CK1, *Aspergillus*-treated hairy roots for 1 day; A_CK4, *Aspergillus*-treated hairy roots for 4 days; Y_CK1, yeast-treated hairy roots for 1 day; Y_CK4, yeast-treated hairy roots for 4 days.

**Figure 12:**
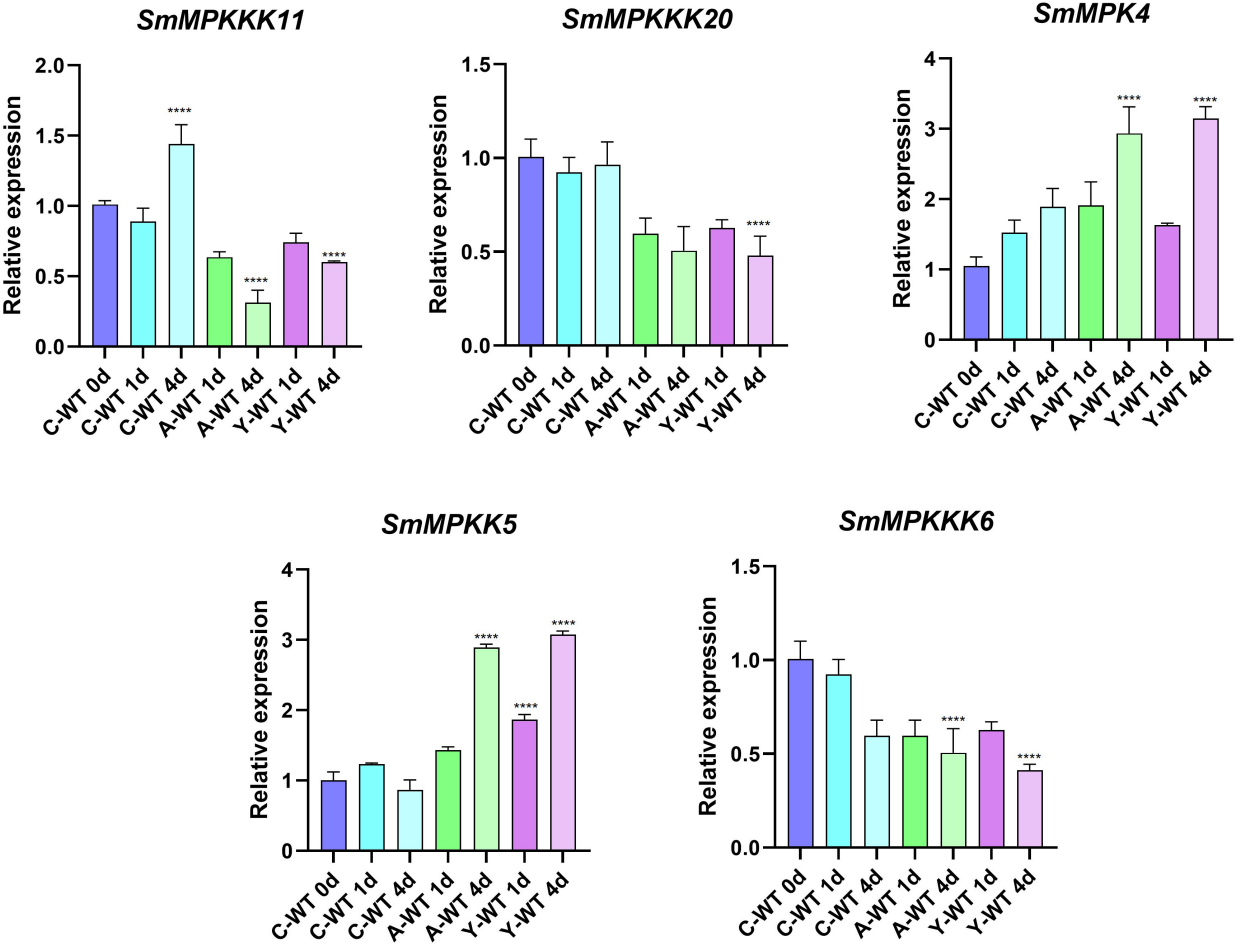
qRT-PCR validation of expression level of candidate SmMAPK genes. Error bars represent the mean ± standard deviation (SD) of three biological replicates. Significant differences were detected with unpaired *t* test. (****) represents a significant difference at the level of p < 0.0001 compared with the control group.

**Figure 13:**
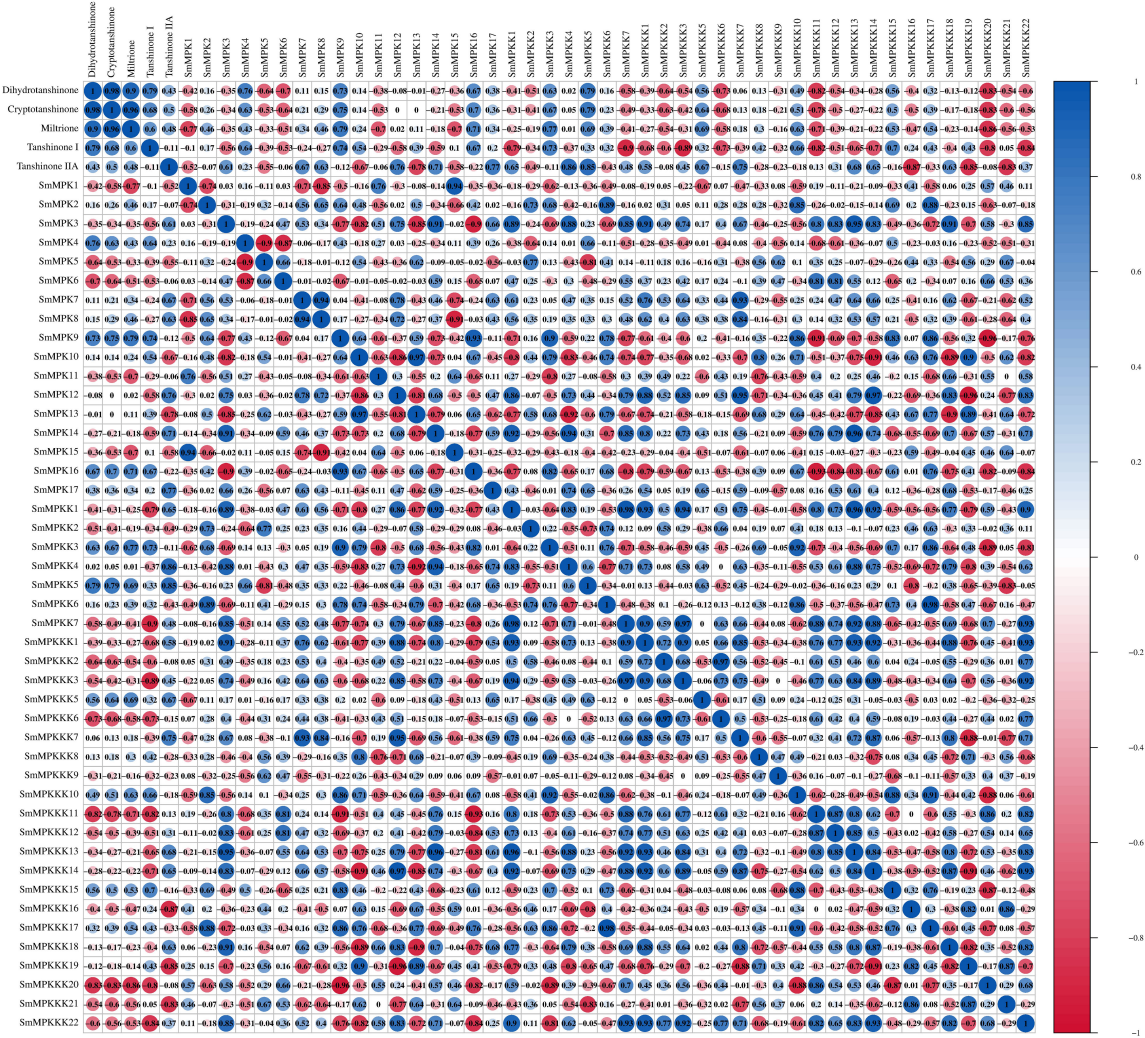
Pearson correlation coefficient analysis to screen the potential SmMAPK genes involved in tanshinone accumulation in *S. miltiorrhiza*. The circle size reflects the strength of the association between the gene expression level and tanshinone content; red and blue circles mean a negative and a positive correlation, respectively. No circles means no correlation.

To identify fungal elicitor-activated SmMAPK genes that may be involved in tanshinone accumulation, hairy roots samples of yeast extract- and *A. niger-*treated groups, together with the control group, were collected before and after 1 and 4 days of treatments, then analyzed by RNA sequencing. All SmMAPK genes were screened in the transcriptome data, and their expression level was visualized using tbtool software to generate heatmaps (**Figures 8**, **11**). Consistent with RNA-seq data, RT-qPCR (**Figure 12**) confirmed the significant upregulation of *SmMPK4* and *SmMPKK5*, while *SmMPKKK6*, *SmMPKKK11*, and *SmMPKKK20* were significantly suppressed in response to fungal elicitation. These results corroborate the reliability of our transcriptomic data and highlight key genes. To better understand the role of MAPK genes in fungal elicitor-mediated tanshinone accumulation, a Pearson correlation coefficient analysis was done. The results revealed that eight MPKs positively correlated with three enhanced tanshinone compounds (dihydrotanshinone, cryptotanshinone, and miltirone) with high negative correlation in *SmMPK9* and *SmMPK16* and high positive correlation of dihydrotanshinone, cryptotanshinone with *SmMPK4*, while 10 MPKs correlated negatively with them, especially *SmMPK1* and *SmMPK6*. Four of the seven identified SmMPKKs have a positive correlation with tanshinones, but only *SmMPKK3* and *SmMPKK5* showed a significant correlation. The other members showed medium-to-low negative correlation. Most SmMPKKK genes negatively correlated with tanshinones, and a very high correlation was observed in *SmMPKKK6*, *SmMPKKK11*, and *SmMPKKK20* (**Figure 13**).

## Discussion

In recent decades, Chinese herbal medicines and phytometabolites have shown health-promoting actions and promising effects in enhancing immunity (Hao and Liu, 2022).

Genome-wide identification of gene families gives valuable information on the regulation process in plants (Wang et al., 2024). SmMAPK genome-wide identification, characterization, and phylogenetic analysis can provide useful information to enhance our understanding of their potential regulatory roles in tanshinone accumulation in *S. miltiorrhiza*. The *A. thaliana* genome contains 20 AtMAPKs, 10 AtMAPKKs, and 80 AtMAPKKKs, while 17 OsMAPKs, 8 OsMAPKKs, and 75 OsMAPKKKs were detected in the rice genome (Rao et al., 2010). In this study, 17 MAPKs, 7 MAPKKs, and 22 MAPKKKs were identified in *S. miltiorrhiza* genome, which were distributed on nine chromosomes, with MWs ranging from 25,860.58 to 95,481.2 Da and theoretical pI ranging from 4.51 to 9.52.

MAPK genes are important in various cellular processes, including development, growth, cell death, and abiotic and biotic stress response (Smékalová et al., 2014; Guan et al., 2014; Wang et al., 2018; Zhang et al., 2017; Cai et al., 2014). A very significant number of stress-responsive and phytohormone elements were detected in the promoter sites of SmMAPK genes, in addition to some development and growth cis-acting elements. That indicates that SmMAPK genes are mostly related to biotic and abiotic stress as well as phytohormone response. Moreover, the identification of 5 defense-response elements in *SmMPK4*, *SmMPK7*, and *SmMPK17* of which *SmMPK4* has an elicitor-mediated activation element (**Figure 4E**), 2 defense elements in *SmMPKK2*, which has 1 elicitor-mediated activation element and 1 defense element in *SmMPKK5* (**Figure 5E**), in addition to 17 defense-response elements, were distributed within *SmMPKKK6*, *SmMPKKK2*, *SmMPKKK14*, *SmMPKKK8*, *SmMPKKK9*, *SmMPKKK11*, *SmMPKKK5*, *SmMPKKK15*, *SmMPKKK20*, *SmMPKKK19*, *SmMPKKK21*, and *SmMPKKK22*, of which *SmMPKKK11* also contains one elicitor-mediated activation element (**Figure 6E**), which suggest these members to be possible candidate genes involved in fungal elicitor-mediated tanshinone accumulation.

The divergence in exon/intron structure is crucial to the evolution of gene families. Thus, the structure of gene exons and introns can support phylogenetic groupings (Shiu and Bleecker, 2003; Zhang et al., 2012; Cao et al., 2011). The structures of the SmMAPK genes were predicted from the *S. miltirrohiza* genome. All SmMAPKs genes from the TDY subgroup have 10 exons, while most TEY subgroup genes have six exons (**Figure 4F**). This high similarity degree was observed among subgroup members and supports their relationships. Similarly, SmMAPKK genes were divided into the following two subgroups: the first subgroup with one exon and the second subgroup with seven to eight exons (**Figure 5F**). In contrast, the number of exons in SmMPKKK genes subgroups varies from one to four exons, while some members have no exons (**Figure 6F**). This large variation in structures may be due to significant changes in the genome during the long evolutionary history.

The protein interaction network analysis (**Figure 7**) revealed that MAPK genes are involved in complex signaling pathways that are likely to play a role in the accumulation of tanshinones in response to fungal elicitors. The interactions with *Arabidopsis* orthologs, particularly those involved in stress and defense responses, suggest that these genes may be key regulators in the signaling pathways that lead to the production of tanshinones.

GO enrichment analysis (**Figure 8**) reveals that the MAPK genes in *S. miltiorrhiza* are involved in a wide range of cellular processes, particularly those related to stress responses, signal transduction, and secondary metabolite biosynthesis. These findings provide valuable insights into the functional roles of these genes and their potential applications in improving tanshinone production.

The KEGG pathway enrichment analysis (**Figure 9**) provides valuable insights into the functional roles of the MAPK genes identified in *Salvia miltiorrhiza*. The results highlight the involvement of these genes in critical signaling pathways, particularly those related to stress responses and plant–pathogen interactions. The enrichment of MAPK genes in both the MAPK signaling pathway and plant–pathogen interaction pathway suggests that these genes are not only involved in stress responses but also play a role in regulating secondary metabolite biosynthesis. The activation of these genes in response to fungal elicitors may lead to the upregulation of biosynthetic pathways involved in tanshinone production. This provides a foundation for further studies aimed at manipulating these genes to enhance tanshinone production through biotechnological approaches.

Tanshinone accumulation in *S. miltiorrhiza* was reported to be significantly induced by fungal elicitors (Zhou et al., 2017; Wu et al., 2022) suggesting that they are a part of defensive mechanisms, especially that tanshinone compounds were reported to have antimicrobial properties. Moreover, cryptotanshinone and dihydrotanshinone I showed higher antimicrobial activity than tanshinone IIA and tanshinone I (Zhao et al., 2011). In our results (**Figure 10C**), both fungal elicitors yeast extract and *A. niger* increased specific tanshinone compound accumulation (miltirone, cryptotanshinone, and dihydrotanshinone I), while no significant increase in tanshinone IIA and tanshinone I was observed. The increase in tanshinone compounds with higher antimicrobial properties further supports that their accumulation was induced as a defensive mechanism. Many studies showed that fungal-responsive MAPK genes have a regulatory role in defensive metabolite accumulation in plants (Kishi-Kaboshi et al., 2010). For example, in *Arabidopsis*, camalexin, the major phytoalexin, is regulated by two fungal-responsive MPK3/MPK6 (Ren et al., 2008). In rice, OsMPK3 and OsMPK6, the orthologs of *Arabidopsis AtMPK3* and AtMPK6, regulate the biosynthesis of momilactones and phytocassanes, the diterpenoid phytoalexins (Kishi-Kaboshi et al., 2010). Interactions between *MKK1*, *MKK4*, *MKK5*, *MAPK3*, and *MAPK6* with *MKK9* in *Brassica rapa* play a major role in camalexin biosynthesis in response to *Alternaria brassicae* (Gaur et al., 2018). The application of yeast extract dramatically increased biphenyl phytoalexins, the defensive metabolites in *Sorbus aucuparia*, by activating the MAPK pathway (Li et al., 2024). Our results (**Figure 11**) revealed upregulation or downregulation of most SmMAPK genes in response to the two fungal elicitors. Moreover, in the Pearson correlation coefficient analysis (**Figure 13**), *SmMPK4* showed a high positive correlation with dihydrotanshinone and cryptotanshinone, the two tanshinones with higher antimicrobial properties. In addition, the promoter analysis of *SmMPK4* (**Figure 4E**) identified two defense-response elements and one elicitor-mediated activation element. Also, the phylogenetic tree (**Figure 3**) showed that *SmMPK4* is the closest ortholog to *Arabidopsis AtMPK4*, a kinase protein involved in defense response and regulation of phytoalexin accumulation (Brodersen et al., 2006; Droillard et al., 2004; Petersen et al., 2000; Schweighofer et al., 2007; Qiu et al., 2008). Overall, these findings indicate *SmMPK4* to be a suitable candidate that has a regulatory role in tanshinone accumulation. *SmMPKK5* has a high positive correlation with both tanshinone compounds and *SmMPK4*. In addition, it has a defense element in the promoter site suggesting that it also may have a regulatory role. *AtMPKK2* is a MAPK kinase required for the activation of *AtMPK4* (Kong et al., 2012). Our results showed that *SmMPKK2*, which is the closest ortholog of *AtMPKK2*, significantly correlated with *SmMPK4*, and its promoter site has two defense elements and one elicitor-mediated activation element. This may suggest its possible regulatory role in tanshinone accumulation. *SmMPKKK6*, *SmMPKKK11*, and *SmMPKKK20* have a very high negative correlation with tanshinone compounds. In addition, the three member promoter sites have defense cis-regulatory elements, and *SmMPKKK11* has one elicitor-mediated activation element suggesting their involvement in fungal elicitor-mediated tanshinone accumulation in *S. miltiorrhiza.*

In conclusion, our findings on the potential roles of MAPK in tanshinone accumulation are consistent with previous studies in *Arabidopsis* and rice, where MAPKs have been shown to regulate the biosynthesis of defensive metabolites such as camalexin and momilactones (Kishi-Kaboshi et al., 2010; Gaur et al., 2018). These similarities suggest a conserved regulatory mechanism across plant species. The identification of key MAPK genes involved in tanshinone biosynthesis opens new avenues for biotechnological applications, such as the use of genetic engineering or fungal elicitors to enhance tanshinone production in *S. miltiorrhiza*.

## Data Availability

The datasets presented in this study can be found in online repositories. The names of the repository/repositories and accession number(s) can be found below: https://bigd.big.ac.cn/gsa/browse/CRA016503, CRA016503.
